# Technologies to investigate the mechanisms of force-based manipulations: a narrative methods review

**DOI:** 10.3389/fpain.2026.1908955

**Published:** 2026-08-28

**Authors:** Arin M. Ellingson, Jennifer A. Bent, Mary F. Barbe, Karl J. Lewis, Simon Y. Tang, Gregory J. Gerling, M. Terry Loghmani, William R. Reed, Greg N. Kawchuk

**Affiliations:** 1 Division of Physical Therapy and Rehabilitation Science, Department of Family Medicine and Community Health, Medical School, University of Minnesota, Minneapolis, MN, United States; 2 Duke University Department of Rehabilitation Services, Duke University Health System, Durham, NC, United States; 3 Department of Rehabilitation Sciences, Medical University of South Carolina, Charleston, SC, United States; 4 Aging + Cardiovascular Discovery Center, Department of Cardiovascular Sciences, Lewis Katz School of Medicine, Temple University, Philadelphia, PA, United States; 5 Meinig School of Biomedical Engineering, Cornell University, Ithaca, NY, United States; 6 Department of Orthopaedic Surgery, Washington University School of Medicine in St. Louis, St. Louis, MO, United States; 7 School of Engineering and Applied Science, University of Virginia, Charlottesville, VA, United States; 8 Department of Graduate Health Professions Physical Therapy Program, Indiana University, Indianapolis, IN, United States; 9 Department of Physical Therapy, University of Alabama at Birmingham, Birmingham, AL, United States; 10 Department of Physical Therapy, Faculty of Rehabilitation Medicine, University of Alberta, Edmonton, Canada

**Keywords:** force-based manipulation, manual therapy, mechanistic research, multi-domain assessment, neurophysiological regulation, non-pharmacological pain treatment, pain modulation, translational pain research

## Abstract

Force-based manipulations (FBM), as defined by the National Center for Complementary and Integrative Health (NCCIH), involve the passive application of mechanical force to the body with therapeutic intent. These approaches are commonly used in pain management, rehabilitation, wellness, and disease prevention, and include spinal and extremity manipulation, mobilization, soft tissue manipulation, massage, myofascial release, osteopathic manipulative treatment, and related manual or instrument-assisted therapies. FBM may involve light touch, pressure, mobilization, thrust, adjustment, needling, or other mechanically mediated inputs, and their effects may be influenced by contextual factors, including interpersonal relational dynamics, physical environment and patient psychophysics. Consistent with ongoing interprofessional efforts to standardize manual therapy nomenclature, this review emphasizes the careful descriptive terminologies and mechanical parameters rather than profession-specific labels. Although these therapeutic approaches are widely used to manage pain and improve function, the biological processes associated with FBM remain unknown and/or inadequately characterized. This narrative review synthesizes established technologies for investigating FBM across multiple domains, including biomechanical loading and motion analysis, tissue, cellular, and molecular imaging, biomarker, transcriptomic, and mechanobiological assessment, neurophysiological monitoring, vascular and microcirculatory assessment, computational modeling/data science, sensory, psychophysical, and contextual assessment, and mechanistic perturbation and neuromodulation methods. Rather than focusing on individual techniques in isolation, this review organizes these methods within a multi-scale framework that relates externally applied mechanical inputs to measurable internal mechanical, neural, vascular, cellular, molecular, regional, and whole-organism responses. Crucially, the physiological impact of these mechanical events is continuously modulated by the operationalized environment and therapeutic alliance, which must be treated as measurable covariates that interact with mechanical inputs. By providing a structured overview of validated and accessible methodologies, this work offers a roadmap for mechanistic FBM research and supports the future development of integrative, interdisciplinary studies. This framework is intended to help investigators characterize applied mechanical inputs using shared descriptive terminology, evaluate physiological responses across levels of structure and function, and examine relationships among mechanical input, contextual factors, biological mechanisms, and clinically meaningful outcomes.

## 1 Introduction

Force-based manipulations (FBM), as defined by the National Center for Complementary and Integrative Health (NCCIH), encompass a broad spectrum of approaches that apply mechanical forces to the body with therapeutic intent (1). These include spinal and extremity manipulation, mobilization, massage, myofascial release, osteopathic manipulative treatment, needling, and other manual or instrument-assisted techniques that mechanically interact with musculoskeletal and related tissues. FBM are widely used to manage pain, improve function, restore mobility, and support rehabilitation across a range of conditions.

Despite their widespread use, the biological and physiological processes associated with FBM remain unclear (2). A central challenge is that FBMs are multi-scale and multi-system interventions. Mechanical input delivered at the body surface is transmitted through tissues, producing tissue deformation/strain and fluid-related mechanical effects that may alter the local mechanical environment. These physical changes may engage peripheral sensory receptors and are associated with responses across neuromuscular, vascular, immune, endocrine, and biochemical systems. As a result, FBM-related processes span spatial scales from molecular and cellular signaling to tissue mechanics, joint motion, whole-body movement, and clinical function, as well as temporal scales from milliseconds during force application to longer-term adaptation and treatment response.

FBM research faces a common challenge across translational biomedical research, where promising mechanistic findings often fail to progress efficiently into clinically meaningful applications (3, 4). As identified by NIH and articulated by Reed et al, the field is hindered by long standing scientific and professional barriers, including fragmented research silos, inaccurate terminology, and a lack of objective multi-scale measures for assessing real-world therapeutic response (1). Addressing these barriers requires a shift from isolated, single domain studies towards an integrated multi-domain framework that captures the pathway from mechanical input to systemic physiological and contextual response.

Importantly, many of the tools required to study these processes already exist. Advances in biomechanics, imaging, neurophysiology, sensory testing, vascular assessment, molecular biology, computational modeling, and data science provide established methods for measuring key components of the FBM response. The primary barrier is not simply a lack of technology, but the absence of an organized methodological framework that links these tools to specific mechanistic questions and supports integration across domains. Without such a framework, FBM research risks remaining fragmented across isolated empirical silos, with limited ability to connect force delivery, tissue behavior, neural activity, vascular response, biochemical signaling, contextual factors, and clinical outcomes.

To move FBM research toward scalable, systems-level data integration, the field requires a shared structure for organizing measurements across biological scales and disciplinary boundaries. Such a structure is needed to support multi-domain study design, advanced computational modeling, machine learning integration, and eventual multi-site clinical implementation registries capable of generating standardized, cross-domain datasets for FBM science.

Here, we summarize established and emerging technologies for investigating the mechanisms of FBM across eight methodological domains. The aims of this narrative methods review are to: (1) operationalize the empirical toolkit by cataloging technologies and identifying the mechanistic questions each is best positioned to answer; and (2) provide a roadmap for future multi-domain studies that link applied mechanical inputs to internal mechanical, neural, vascular, biochemical, sensory, contextual, and clinical responses. This review is intended to provide researchers across biomechanics, neurophysiology, imaging, molecular biology, computational science, and clinical investigation with a shared language and organizational structure for coordinated inquiry into the mechanisms of FBM. Importantly, while this review catalogs a robust cross-section of validated technologies, it is not an exhaustive inventory. Rather, it serves as a foundational starting point reflecting the current methodological landscape, intended to help investigators identify available tools while illuminating methodological gaps and future areas for consideration.

## 2 Conceptual framework for multi-domain FBM research

This review is grounded in a multi-scale view of how FBM interact with biological systems and expands on the FBM model developed by the NCCIH Force-Based Manipulations Research Network (5). Rather than treating FBM as isolated therapeutic techniques or single-system interventions, we conceptualize them as externally applied mechanical inputs that can be measured, experimentally varied, and related to biological responses across multiple levels of organization (6).

At its core, FBM can be viewed as a system-level perturbation. Mechanical input delivered at the body surface is transmitted through skin, fascia, muscle, tendon, ligament, joint, neural, and vascular tissues, producing local tissue deformation/strain, pressure gradients, and fluid-related mechanical effects. These mechanical events may influence the local tissue environment, activate peripheral receptors, and contribute to responses across peripheral, spinal, and supraspinal pathways. They may also be accompanied by changes in motor output, autonomic regulation, metabolism, inflammation, tissue perfusion and remodeling, pain perception, and clinical function.

**Figure 1:**
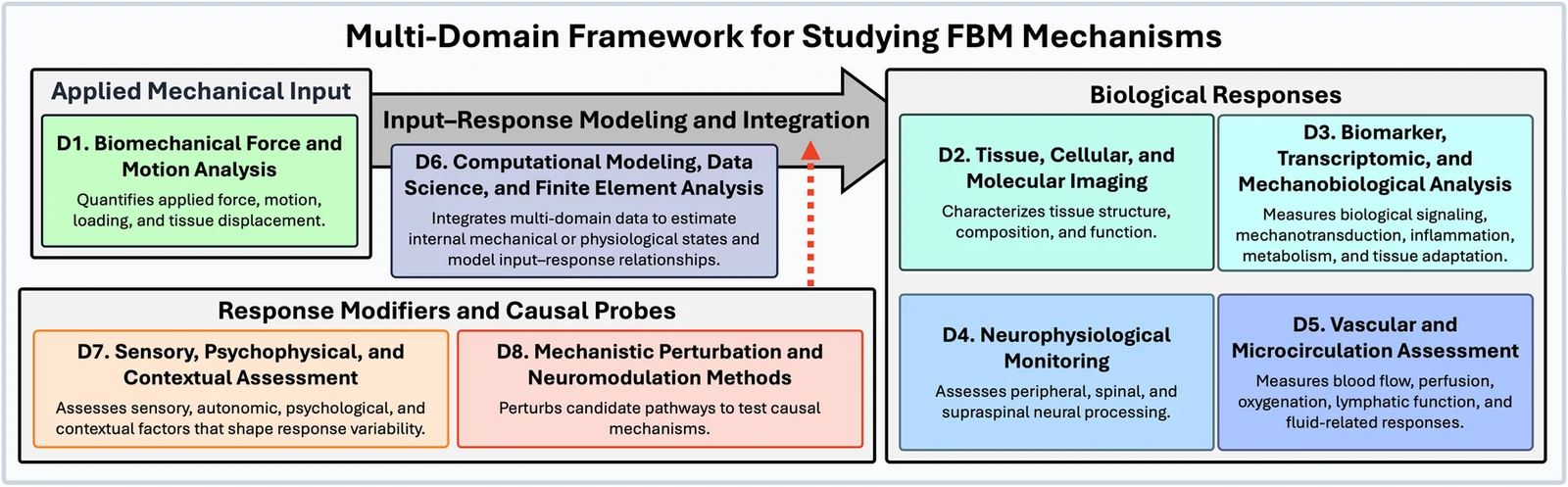
Integrated multi-domain framework for studying mechanisms of force-based manipulation. Force-based manipulation (FBM) is conceptualized as an externally applied mechanical input that can be characterized using Domain 1 methods and related to downstream tissue, molecular, neural, vascular, and physiological responses measured across Domains 2–5. The left-to-right arrow depicts the conceptual progression from applied mechanical input toward downstream biological responses; it does not prescribe a required sequence of study design. Domain 6 supports integration across the pathway through computational modeling, data science, and finite element analysis, including estimation of internal mechanical or physiological states and modeling of input–response relationships. Its position along the arrow reflects this integrative role rather than a distinct biological stage of force transmission. Domain 7 captures sensory, psychophysical, autonomic, psychological, relational, and environmental factors that may modify responses at multiple points. Domain 8 includes mechanistic perturbation and neuromodulation methods used to test candidate pathways and strengthen causal inference. Although the mechanistic pathway is directional, investigators may enter the framework at different points according to the clinical or mechanistic question. Together, the eight domains provide a coordinated methodological framework for relating applied mechanical inputs to biological, contextual, and clinically meaningful responses. A searchable hierarchical version of the framework, including representative methods and supporting citations, is provided as Supplementary Material.

Mechanistic inference in FBM research requires coordinated measurement across domains, as depicted in Figure 1. The left-to-right pathway represents the conceptual progression from an applied mechanical input through tissue-level effects and associated biological responses toward clinically meaningful outcomes (Domains 1–5). Domain 6 supports integration and modeling across this pathway. While this review organizes the domains along a directional input-response pathway to catalog methodological resources, the reality of FBM is highly dynamic. Domain 7 captures the sensory, psychological, and contextual factors that interact with the mechanistic pathway and may modify responses at multiple points. Domain 8 provides approaches for perturbing candidate pathways and strengthening causal inference.

Although the mechanistic input–response pathway is directional, scientific inquiry may begin at different points within the framework. Bottom-up studies may begin with a controlled mechanical input and examine its transmission through tissues, biological encoding, physiological consequences, and relationship to clinical outcomes. Top-down studies may instead begin with a clinical observation—such as a response phenotype, symptom pattern, treatment failure, or variability in treatment response—and use that observation to prioritize candidate mechanisms, methodological domains, and experimental perturbations.

These approaches are complementary and iterative rather than competing. Bottom-up studies can establish biological plausibility and characterize candidate pathways under controlled conditions, whereas top-down studies help anchor mechanistic questions to clinically meaningful observations and sources of response heterogeneity. Clinical observations may generate mechanistic hypotheses; multi-domain studies can test those hypotheses; computational and causal approaches can integrate findings across scales; and the resulting evidence can refine clinical phenotyping, intervention delivery, outcome selection, and subsequent investigation.

The eight domains should therefore be viewed as complementary methodological resources rather than fixed sequential stages. Investigators may enter the framework at different points and select domains according to the clinical or mechanistic question, relevant biological and temporal scales, maturity of the evidence, and intended translational application. To support practical use of the framework, an interactive supplementary taxonomy presents the domains, subsections, representative method families, and supporting citations in an expandable, searchable format.

The eight domains described in this review organize these methodological capabilities according to the components of FBM mechanisms they are best positioned to measure:

**Domain 1: Biomechanical Force and Motion Analysis** characterizes applied mechanical inputs, movement behavior, joint motion, tissue displacement, and loading patterns.
**Domain 2: Tissue, Cellular, and Molecular Imaging** quantifies structural, compositional, and functional characteristics of tissues across *in vivo* and *ex vivo* scales.
**Domain 3: Biomarker, Transcriptomic, and Mechanobiological Analysis** assesses biological signaling, mechanotransduction, inflammation, metabolism, and tissue adaptation.
**Domain 4: Neurophysiological Monitoring** evaluates how mechanical inputs are encoded and processed across peripheral, spinal, and supraspinal nervous system pathways.
**Domain 5: Vascular and Microcirculation Assessment** measures blood flow, perfusion, oxygenation, lymphatic function, and fluid-related physiological responses.
**Domain 6: Computational Modeling, Data Science, and Finite Element Analysis** integrates multi-domain measurements and estimates internal mechanical or physiological states that are difficult or impossible to measure directly.
**Domain 7: Sensory, Psychophysical, and Contextual Assessment** characterizes sensory processing, pain modulation, autonomic reactivity, psychological state, expectations, real-world symptom dynamics, and other contextual factors that shape individual response.
**Domain 8: Mechanistic Perturbation and Neuromodulation Methods** provide tools for perturbing specific neural, sensory, vascular, or biomechanical components of the system to strengthen causal inference. Whereas Domain 1 characterizes the mechanical input and resulting motion or deformation, Domain 8 experimentally manipulates candidate system components to determine whether altering them changes the FBM response.

**Figure 2:**
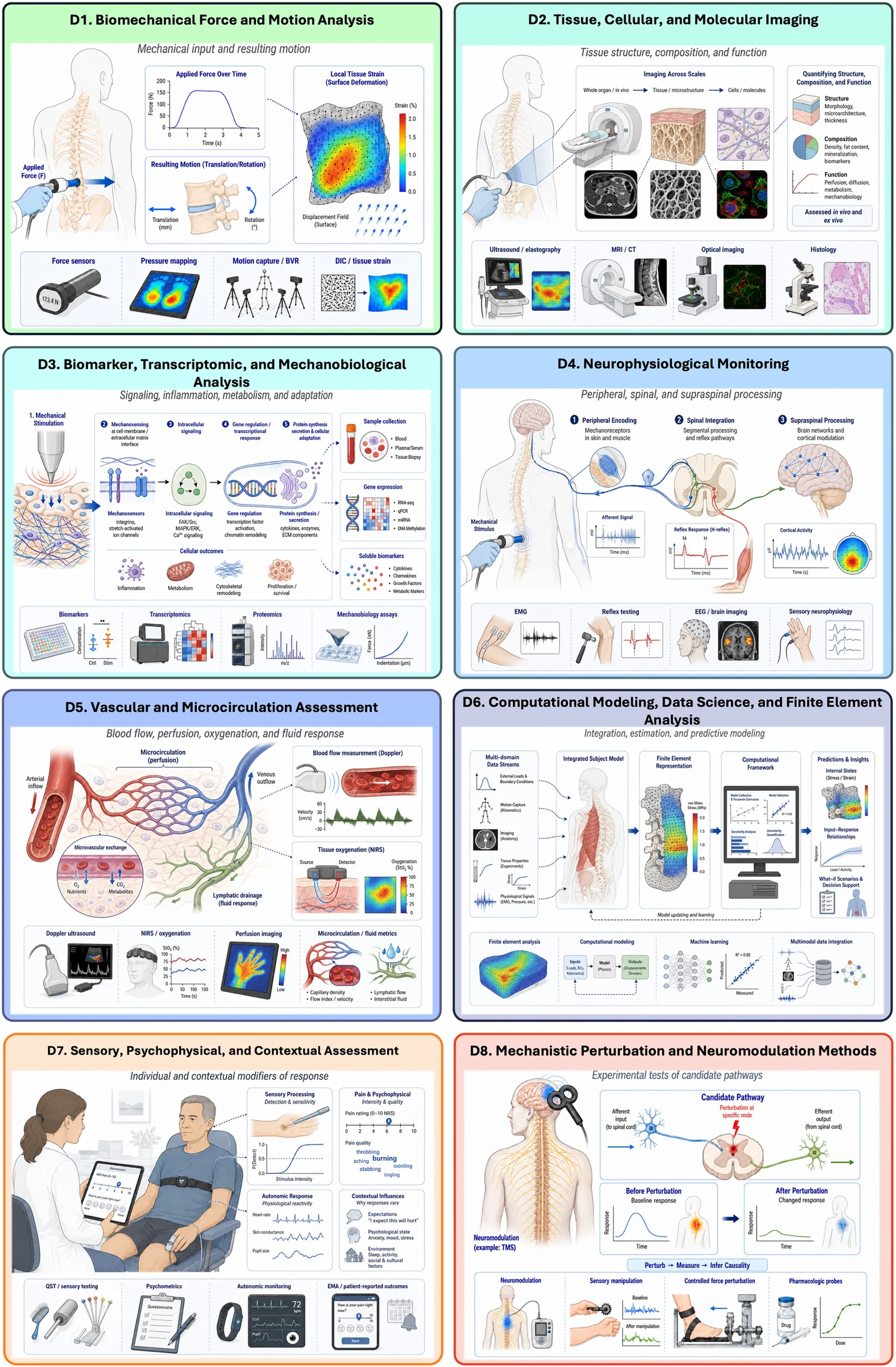
Representative methodological approaches across the eight domains of force-based manipulation research. Each panel illustrates the primary measurement focus and representative technologies associated with one methodological domain: (D1) biomechanical force and motion analysis; (D2) tissue, cellular, and molecular imaging; (D3) biomarker, transcriptomic, and mechanobiological analysis; (D4) neurophysiological monitoring; (D5) vascular and microcirculation assessment; (D6) computational modeling, data science, and finite element analysis; (D7) sensory, psychophysical, and contextual assessment; and (D8) mechanistic perturbation and neuromodulation methods. The examples shown are illustrative rather than exhaustive. Detailed descriptions, applications, and supporting citations are provided in the corresponding manuscript sections and interactive supplementary hierarchy.

Figure 2 provides a visual overview of the primary measurement focus and representative methodological approaches associated with each of the eight domains. Together, these domains provide a practical framework for designing studies that move beyond single-modality outcomes toward integrated mechanistic investigation. The purpose of this structure is not to reduce FBM mechanisms to one pathway, but to help researchers identify what each method can measure, what mechanistic question it can address, and what complementary measurements are needed to link externally applied mechanical forces to multi-system biological responses and clinically meaningful outcomes.

## 3 Methodological domains for FBM research

### 3.1 Domain 1: biomechanical force and motion analysis

This domain provides the foundational tools for characterizing the mechanical inputs delivered during FBM and quantifying how those inputs are expressed as movement, loading, tissue displacement, and regional or joint-level mechanical behavior (7–9). These methods define what force was applied, how it was delivered, and how the body mechanically responded. As such, biomechanical force and motion analysis provides the entry point for linking FBM delivery parameters to downstream tissue, neural, vascular, biochemical, and clinical responses.

#### 3.1.1 D1.1 applied force quantification

Instrumented force measurement is essential for characterizing FBM delivery. Relevant parameters include force magnitude, direction, rate of loading, duration, contact area, dosage, and delivery method. Force plates, pressure sensitive resistors, precision force gauges, and instrumented tables or treadmills can quantify ground reaction forces and loading patterns during treatment or treatment-related positioning (10–15), while dynamometry, including handheld, isokinetic, grip-based, and task-specific systems, can quantify muscular force generation, joint torque, provider-applied force, and participant resistance (16–21). Arthrometry provides measures of joint laxity and stiffness under applied loads, serving as functional proxies for passive mechanical behavior (22).

Instrumented manual therapy devices provide a more direct approach for quantifying FBM delivery. For example, quantifiable soft tissue manipulation (QSTM) systems have been developed to capture objective force-motion parameters during manual therapy, including both localizing and dispersive patterns of soft tissue loading (23, 24). These approaches illustrate how FBM delivery can be translated from a clinician-applied manual procedure into measurable input variables, supporting more reproducible dosing, technique comparison, and linkage between applied forces and biological responses.

Robotic and haptic systems introduce additional precision and reproducibility into force application and measurement. Instrumented orthoses, exoskeletons, and robotic platforms equipped with force and torque sensors allow controlled variation of input parameters, including magnitude, direction, rate, and duration (25). Shear and torsional force platforms can capture multidirectional loading conditions relevant to rotational or shear components of FBM, while haptic interfaces provide standardized approaches for probing tissue mechanical response under controlled loading conditions.

#### 3.1.2 D1.2 *in vivo* motion analysis

Motion tracking technologies quantify movement across multiple levels of resolution, supporting assessment of coordination, intersegmental motion, and movement variability. Optical motion capture systems using reflective markers and multi-camera arrays remain widely used for whole-body kinematic analysis, allowing calculation of joint angles, segment coordination, and task-specific movement strategies (26–30). These measures can be compared across conditions to evaluate how movement behavior differs before, during, or after FBM. Related high-fidelity spine kinematic approaches, including work quantifying aberrant neck motion, further demonstrate how motion analysis can identify clinically relevant movement patterns that may inform FBM mechanism studies (31).

Biplane videoradiography (BVR) extends motion analysis to high-precision skeletal tracking by enabling direct measurement of bone motion *in vivo* with excellent accuracy (32–34). This technique is particularly suited for quantifying intervertebral and intersegmental kinematics, including coupled motion, instantaneous axes of rotation, and cycle-to-cycle variability (35, 36). BVR has been used to quantify cervical intervertebral kinematics associated with neck pain (37), as well as before, during, and after high-velocity low-amplitude manipulation, providing a direct example of how an externally applied manipulation can be linked to segment-level spinal motion (38).

Emerging markerless motion tracking approaches, enabled by computer vision and machine learning, provide scalable alternatives for capturing movement in less constrained environments (39–42). Inertial measurement units (IMUs) and electromagnetic tracking systems offer portable options for field-based and clinical assessments, enabling kinematic data collection during naturalistic movement or ambulatory monitoring (43–45). These tools are particularly relevant for evaluating whether FBM-related changes observed in laboratory settings translate to movement behavior during daily activities, although direct FBM-specific ambulatory applications remain less developed than laboratory-based motion analysis.

#### 3.1.3 D1.3 tissue deformation

At the tissue level, indentation testing, compliance testing, and digital image correlation (DIC) quantify local tissue displacement and deformation under applied loading conditions. DIC uses high-resolution optical tracking of surface speckle patterns to resolve full-field displacement and strain maps, providing a means to characterize how mechanical inputs propagate through superficial and underlying tissue layers (46, 47). Skin and soft-tissue deformation modeling can further describe relative motion between superficial and deeper structures, helping bridge externally measured force parameters with internal tissue-level mechanical behavior.

Strain-based approaches are particularly relevant for soft tissue FBM because they can quantify regional mobility, asymmetry, and mechanical behavior at or near the tissue surface. For example, strain-based biomarkers measured at the skin surface have been proposed to differentiate asymmetries in soft tissue mobility associated with myofascial pain (46). Such methods provide a concrete example of how tissue deformation measures can move beyond descriptive palpation toward quantitative assessment of soft tissue mechanical behavior relevant to manual therapy targets.

Haptic interfaces provide additional standardized approaches for probing tissue mechanical response (48, 49). These methods can be used to apply controlled FBM-like loading conditions in laboratory settings and to evaluate how variation in applied force, contact geometry, or tissue state influences local mechanical response.

#### 3.1.4 D1.4 ambulatory monitoring and wearable kinematics

Wearable sensor systems extend biomechanical measurement beyond the laboratory into clinical and real-world environments. IMU-based systems enable continuous monitoring of movement patterns, postural behavior, and physical activity in free-living conditions, supporting assessment of how FBM-related changes in movement strategies are expressed during daily activities. Wearable and ambulatory systems may be particularly useful for determining whether short-term changes in pain, mobility, or movement coordination following FBM persist outside the laboratory and relate to real-world function.

At present, the strongest FBM-specific biomechanical examples remain laboratory-based measures of applied force, *in vivo* motion, and tissue deformation. Ambulatory monitoring should therefore be framed as an important extension of these methods rather than an established FBM evidence base. When paired with patient-reported outcomes, ecological momentary assessment, or clinical performance measures, wearable kinematic systems may help bridge mechanistic laboratory findings with patient-centered function across the diverse physical environments of daily life.

Together, these methods characterize both the externally applied mechanical input and the resulting motion, loading, and tissue-level mechanical behavior. When integrated with imaging, neurophysiological, vascular, molecular, sensory, and clinical outcomes, Domain 1 methods provide the mechanical foundation needed to evaluate how variation in FBM delivery relates to downstream biological and functional responses.

### 3.2 Domain 2: tissue, cellular, and molecular imaging

This domain includes *in vivo* and *ex vivo* imaging methods used to visualize the structural, compositional, and functional state of tissues relevant to FBM (50, 51). These approaches characterize tissue morphology, alignment, composition, stiffness, motion, perfusion, and microscopic architecture across scales ranging from cellular organization to whole-body structure. In the context of FBM, imaging methods help define the biological substrate through which mechanical forces are transmitted and provide measurable outcomes that can be related to force delivery, movement, neurophysiological responses, and clinical change.

#### 3.2.1 D2.1 ultrasound imaging and elastography

Ultrasound provides real-time, non-ionizing visualization of soft tissues, including muscle, tendon, ligament, fascia, peripheral nerves, and superficial joint structures. B-mode ultrasound enables assessment of tissue morphology, thickness, displacement, and motion during or following externally applied loading. Speckle-tracking ultrasound extends this capability by quantifying tissue displacement and strain patterns, supporting analysis of how mechanical inputs are expressed within layered tissues (52).

Ultrasound elastography, including strain and shear-wave elastography, provides quantitative estimates of tissue stiffness and viscoelastic behavior (53–55). These methods are directly relevant to FBM because they can quantify tissue mechanical properties before and after manual or instrument-assisted loading. For example, ultrasound and elastography have been used to visualize deep fascia changes following fascial manipulation in a case study (56), quantify stiffness and thickness changes in the thoracolumbar fascia and lumbar erector spinae following a myofascial technique in adults with chronic low back pain (57), assess changes in plantar fascia and intrinsic foot muscle stiffness following instrument-assisted soft tissue mobilization (58), and evaluate masseter muscle stiffness before and after massage using shear-wave elastography (59). These studies illustrate how ultrasound-based measures can be used to evaluate FBM-related changes in tissue stiffness, fascial mobility, and regional soft-tissue mechanical behavior—making them informative measures for microvascular and hemodynamic mechanisms discussed in Domain 5.

Doppler ultrasound methods, including color, power, and spectral Doppler, characterize vascular flow direction and velocity, while contrast-enhanced ultrasound (CEUS) and vector flow imaging can provide spatially resolved measures of perfusion and flow behavior (60, 61). Although vascular physiology is addressed more directly in Domain 5, ultrasound-based vascular imaging is included here as an imaging modality that can link local tissue mechanics with hemodynamic response. Direct FBM applications of CEUS and advanced vascular ultrasound remain less developed than elastography-based soft tissue studies, but these methods are well positioned to evaluate whether manual or instrument-assisted loading alters local perfusion or microvascular behavior.

#### 3.2.2 D2.2 advanced MRI techniques

Magnetic resonance imaging (MRI) provides a comprehensive platform for assessing structural, compositional, and functional characteristics of deeper tissues relevant to FBM. Conventional T1- and T2-weighted sequences enable detailed visualization of intervertebral discs, ligaments, cartilage, fascia, peripheral nerves, and paraspinal musculature (62). These structural images can characterize baseline tissue state, pathology, or anatomical features that may influence force transmission and treatment response.

Quantitative MRI techniques, including T1 and T2 mapping, T1ρ, ultrashort echo time (UTE), PETRA, dGEMRIC where applicable, and Dixon-based fat fraction imaging, provide measures related to tissue hydration, proteoglycan content, collagen-associated signal behavior, and fatty infiltration (63–67). These methods are particularly relevant for evaluating tissue composition longitudinally or across experimental conditions. In FBM research, quantitative and structural MRI may serve two complementary roles: measuring treatment-associated changes when used in longitudinal designs, and informing FBM procedure selection or interpretation by characterizing baseline tissue morphology, composition, degeneration, pathology, or response to loaded positions (68, 69). For example, qMRI studies of intervertebral disc structure, morphology, and biochemical composition can help define the tissue substrate through which FBM forces are transmitted and may identify tissue states that influence mechanical response or treatment candidacy.

Diffusion-based MRI methods, including diffusion tensor imaging (DTI) and diffusion spectrum imaging (DSI), characterize water diffusion behavior and microstructural organization in neural and connective tissues (70, 71). Magnetic resonance elastography (MRE) provides estimates of tissue stiffness in deeper anatomical regions, complementing ultrasound elastography for tissues not accessible by surface-based methods (72–74).

MRI can also be used to assess neural and vascular function. Functional MRI (fMRI; BOLD) and perfusion MRI, including arterial spin labeling (ASL), provide measures of regional hemodynamic activity. In contrast to structural and quantitative MRI approaches that often characterize baseline tissue state, fMRI has been used more directly to measure FBM-associated effects on central nervous system processing (75–78). Examples include studies evaluating cerebral hemodynamic responses to pain following thoracic thrust manipulation (79), resting-state functional connectivity after manual therapy in induced low back pain (80), Neurologic Pain Signature activation following thoracic spinal manipulation (77), and brain mechanisms or connectivity changes associated with manual therapy in chronic low back pain (81, 82). In this review, these studies are included here as examples of MRI-based effect measurement, while their interpretation as supraspinal neural response measures is addressed primarily in Domain 4.

#### 3.2.3 D2.3 radiographic imaging

X-ray-based imaging provides structural and alignment information relevant to mechanical behavior at regional and whole-body scales. Projection radiography enables assessment of skeletal alignment, posture, and degenerative or structural features that may influence force transmission. Fluoroscopy provides dynamic visualization of joint or segmental motion and can be used to assess movement during functional tasks or controlled mechanical loading (83).

Computed tomography (CT) and cone-beam CT (CBCT) provide high-resolution three-dimensional imaging of bone geometry, supporting structural analysis, anatomical measurement, and subject-specific model generation for computational applications (84). EOS imaging enables low-dose, weight-bearing assessment of whole-body alignment, allowing evaluation of posture and skeletal configuration under physiological loading conditions. Dual-energy x-ray absorptiometry (DXA) provides measures of bone mineral density and body composition, which may be relevant when characterizing the structural context in which FBM forces are applied.

Nuclear imaging approaches, including single-photon emission computed tomography (SPECT) and positron emission tomography (PET), provide spatially resolved measures of metabolic or molecular activity and may be useful for evaluating inflammation, bone turnover, neural metabolism, or other tissue processes in relation to FBM (85–88). PET imaging has been used directly in FBM contexts, including studies of cerebral metabolic changes after chiropractic spinal manipulation for neck pain (50) and glucose metabolic changes in the brain and muscles of patients with nonspecific neck pain treated with spinal manipulation therapy (89). These examples illustrate how metabolic imaging can extend FBM research beyond structure alone toward physiological and molecular activity.

#### 3.2.4 D2.4 histologic and microscopic imaging

*Ex vivo* histologic and microscopic imaging approaches extend tissue characterization to cellular and subcellular scales. Histology, immunohistochemistry, immunofluorescence, confocal microscopy, multiphoton microscopy, and electron microscopy enable visualization of tissue architecture, cellular organization, extracellular matrix structure, inflammatory cell presence, fibrosis, and matrix remodeling (8, 90, 91). These methods provide morphological context for interpreting tissue-level adaptation following mechanical loading or FBM-like interventions.

Within this domain, *ex vivo* approaches are included primarily as imaging platforms for visualizing structural, morphological, and compositional tissue changes in response to FBMs. For example, histological/immunohistological staining and then microscopy have been used to show that there are reductions in myofiber damage after massage-like cyclic compressive loading, and reductions in inflammatory cell infiltration into tissues, or reductions in matrix proteins after cyclic compressive loading or manual therapy, for example (92–94). Molecular signaling, mechanotransduction pathways, gene expression, cytokine responses, and other biochemical assays derived from these models are addressed in Domain 3. This distinction is important because microscopy and histology can show where structural tissue changes occur, whereas biomarker, transcriptomic, and mechanobiological assays are needed to determine what biological processes may be driving those changes.

Together, imaging methods define the anatomical, compositional, functional, and metabolic context in which FBM occurs. When interpreted alongside mechanical input, movement, neurophysiological activity, vascular response, molecular signaling, and clinical outcomes, imaging provides a critical bridge between externally applied forces and measurable biological tissue states (95).

### 3.3 Domain 3: biomarker, transcriptomic, and mechanobiological analysis

This domain includes methods used to quantify biological signaling and cellular responses associated with FBM-related mechanical loading. In contrast to Domain 2, which focuses on visualization of tissue structure and morphology, Domain 3 focuses on measurable biochemical, molecular, genetic, epigenetic, and cellular signaling responses (96–98). These approaches help characterize how mechanical stimuli may be transduced into biological activity, inflammatory or metabolic change, tissue remodeling, and longer-term adaptation.

#### 3.3.1 D3.1 systemic and tissue biochemical marker assays

Biochemical and molecular assays quantify downstream biological variables associated with cellular activity, tissue adaptation, and systemic response to FBM. Enzyme-linked immunosorbent assays (ELISA), Western blotting, multiplex immunoassays, and related protein-based methods can measure cytokines, growth factors, extracellular matrix proteins, tissue turnover markers, neuropeptides, and mechanosensitive signaling molecules (99–104). These measures can be compared across experimental conditions or time points to characterize biological profiles associated with FBM exposure.

Manual therapy animal models provide some of the clearest examples of how biochemical and histologic markers can be used to evaluate FBM-related biological effects. In a rat model of repetitive strain injury, assays of systemic and tissue biomarkers have been used to determine that preventive manual therapy can reduce the production of biochemical markers of inflammation, fibrosis, and tissue remodeling, including repetitive strain-induced serum biomarkers of inflammation and fibrosis (10, 105) and fibrogenic proteins in neuromuscular tissues (106). Another study found decreased inflammation and rescue of muscle loss using instrumentation-assisted soft tissue manipulation in a rodent hindlimb unloading model (107). These studies illustrate how cytokine, extracellular matrix, and fibrosis-related outcomes can provide mechanistic evidence linking externally applied manual loading to biological tissue responses.

Microdialysis enables *in vivo* sampling of metabolites and soluble mediators within local tissues, providing time-resolved measures of the extracellular biochemical environment (108, 109). This approach may be useful for assessing inflammatory mediators, neuropeptides, metabolic byproducts, or local tissue responses before and after mechanical intervention. Serum, plasma, saliva, or tissue biomarker panels can provide broader measures of systemic inflammation, tissue remodeling, neuroendocrine response, and stress physiology, including markers such as IL-6, TNF-α, substance P, CGRP, collagen degradation products, cortisol, and oxytocin (110, 111). These systemic markers do not identify FBM mechanisms in isolation, but they can help characterize whole-organism biological response when paired with mechanical, imaging, neurophysiological, sensory, and clinical outcomes—particularly because markers of stress physiology (cortisol, oxytocin) are highly responsive to the physical and relational clinical environment (112).

#### 3.3.2 D3.2 transcriptomic, proteomic, metabolomic, and epigenetic responses

Gene-level analyses, including quantitative PCR, bulk RNA sequencing, and single-cell RNA sequencing, quantify changes in gene expression associated with cellular responses to mechanical loading (113–116). These approaches can identify transcriptional pathways involved in inflammation, extracellular matrix remodeling, neuronal signaling, immune regulation, and mechanotransduction. Single-cell approaches may be particularly useful for identifying cell-specific responses that are obscured in whole-tissue analyses.

Proteomic and metabolomic profiling extend this analysis by characterizing broader molecular signatures associated with FBM-relevant mechanical stimulation. Proteomics can identify changes in protein expression, post-translational modification, and signaling network activity, while metabolomics can characterize shifts in energy metabolism, oxidative stress, inflammatory metabolism, or tissue repair processes. Epigenetic analyses, including DNA methylation profiling, chromatin accessibility assays, and histone modification assays, provide measures of regulatory changes that may contribute to longer-term adaptation following repeated mechanical input.

At present, direct transcriptomic, proteomic, metabolomic, and epigenetic applications in FBM research remain less developed than biomarker and histologic approaches. However, the manual therapy and mechanotherapy models described above provide experimental platforms in which these methods could be applied to determine whether repeated mechanical input produces cell-type-specific transcriptional programs, protein-level signaling changes, metabolic shifts, or epigenetic regulation related to tissue remodeling, inflammation resolution, or nociceptive processing. These approaches are particularly important for moving FBM research from descriptive tissue outcomes toward pathway-level understanding of biological response of musculoskeletal tissues.

#### 3.3.3 D3.3 mechanobiological signaling and cellular response assays

Mechanobiological and cellular response assays provide measures of how cells sense and respond to mechanical stimuli at the molecular and cellular levels. Mechanosensitive ion channels, including Piezo1, Piezo2, and TRPV4, serve as primary transducers of mechanical stimuli at the cell membrane, converting physical deformation into intracellular calcium signals and downstream signaling cascades. Piezo2, in particular, is expressed in proprioceptive neurons and Merkel cells, making it a candidate mediator of the sensory encoding of FBM-related mechanical inputs.

Mechanosensitive transcriptional regulators, including YAP, TAZ, and focal adhesion kinase (FAK), integrate mechanical signals from the extracellular matrix into gene expression programs governing cell proliferation, differentiation, inflammatory signaling, and extracellular matrix remodeling (117). Conceptual work in mechanotherapy has emphasized that physical therapists and manual therapy researchers can use controlled mechanical loading as a tool to influence musculoskeletal repair, adaptation, and regenerative processes (118–121). Within FBM-relevant experimental models, connective tissue stretch has been shown to reduce soluble TGF-β1 and type I procollagen in mouse subcutaneous connective tissue (121), while stretching has also been shown to influence inflammation resolution in connective tissue (120). These studies support the broader premise that externally applied mechanical inputs can regulate biochemical pathways relevant to fibrosis, inflammation, and tissue remodeling.

Modeled manual therapy studies provide direct examples of how FBM-like loading can be used to test mechanobiological hypotheses. Modeled manual therapy has been shown to attenuate postoperative adhesions (122), and manual therapy in repetitive task models has been shown to prevent nociceptor activity, sensorimotor dysfunction, and neural fibrosis (123). These findings illustrate how mechanical input can be linked to tissue remodeling, neural tissue fibrosis, and nociceptive signaling, providing a bridge between cellular/tissue biology and neurophysiological mechanisms (10, 105, 106, 123).

Massage-like loading studies provide complementary mechanotherapy analogs for understanding dose, timing, and tissue adaptation (124–127). Cyclic compressive loading has been shown to facilitate recovery after eccentric exercise (93), massage-like compressive loading has been shown to improve passive mechanical properties and recovery of active muscle properties in animal models (127–129), and massage has been shown to enhance skeletal muscle regrowth and remodeling in both massaged and contralateral non-massaged limbs (126). Although these studies are not all clinical FBM trials, they provide mechanistically relevant evidence that controlled mechanical loading can influence inflammation, repair, muscle remodeling, and systemic adaptation.

Structural assays such as histology, immunohistochemistry, immunofluorescence, and electron microscopy can be used alongside and in parallel with these molecular methods to localize biological signaling within specific tissues, regions, or cell populations. Such structural assays can contextualize molecular and cellular findings within tissue-level adaptation. When integrated with imaging, biomechanics, neurophysiology, vascular assessment, sensory testing, and computational modeling, biomarker and mechanobiological approaches help connect externally applied mechanical forces to cellular mechanotransduction, tissue remodeling, inflammation resolution, and potential biological mechanisms of FBM response.

### 3.4 Domain 4: neurophysiological monitoring

This domain includes electrophysiological and neuroimaging methods used to characterize how FBM-related mechanical inputs are encoded by peripheral receptors, processed within spinal circuits, and integrated across supraspinal networks (130–134). These approaches provide measures of neural and neuromuscular activity that may link mechanical stimulation to changes in motor output, sensory processing, pain modulation, and autonomic regulation (135). Foundational reviews of spinal manipulation neurophysiology have emphasized that FBM may influence primary afferent activity, spinal reflex excitability, motor control, and pain-processing systems, although the specificity and clinical interpretation of these effects depend strongly on study design and measurement approach (136–140). In FBM research, neurophysiological methods are particularly important because applied force parameters may shape afferent input, while contextual factors such as arousal, expectation, prior pain experience, and psychological state may influence spinal and supraspinal processing.

#### 3.4.1 D4.1 peripheral and spinal excitability

Electromyography (EMG) provides a primary method for quantifying motor system output (98, 141–147). Surface EMG enables non-invasive assessment of muscle activation patterns, including timing, amplitude, and coordination across muscle groups. In FBM research, EMG has been used to evaluate neuromuscular responses during or following spinal manipulation, including studies linking SMT force characteristics to paraspinal muscle activity (148), examining treatment and response factors in muscle activation during spinal manipulation (149), and comparing manipulation and mobilization with respect to vertebral displacement and muscle response patterns (150). These studies illustrate how EMG can connect externally applied mechanical inputs to neuromuscular output.

High-density surface EMG extends this capability by enabling spatial mapping and decomposition of motor unit activity, providing estimates of motor unit recruitment patterns, discharge behavior, and spatial activation distribution (151). For example, spinal manipulation has been examined using motor unit behavior approaches, supporting the use of higher-resolution EMG methods to evaluate whether FBM alters recruitment strategies or motor unit discharge characteristics (152). Intramuscular EMG enables recording from deeper or segmental musculature not accessible through surface methods, supporting more localized assessment of neuromuscular activity in regions associated with joint stability or segmental control (153).

Complementary techniques such as mechanomyography and tensiomyography provide measures of the mechanical response of muscle contraction, offering additional characterization of contractile behavior and muscle mechanical properties (154–157). Nerve conduction studies assess peripheral nerve transmission properties, including conduction velocity, latency, and signal amplitude, providing information on nerve function and integrity (158–161), while teased nerve fiber electrophysiology can show the absence or presence of aberrant ongoing activity in nerve fibers [which is indicative of nerve damage (123)]. Such electrophysiological testing methods have been used to show that manual therapy can improve median nerve function and integrity, helping to explain why patients with carpal tunnel syndrome can present with improved functional status after massage therapy treatment (162, 163).

Spinal reflex and segmental processing can be assessed using H-reflex and F-wave methodologies (164–167). The H-reflex serves as an electrophysiological analog of the monosynaptic stretch reflex and is commonly used to estimate reflex excitability and presynaptic modulation within spinal circuits. These measures have been used directly in spinal manipulation research, including studies reporting short-term changes in H-reflex amplitude following spinal manipulation (167), comparative studies of cervical and lumbar manipulation on spinal reflex excitability (168), and H-reflex/V-wave assessments following spinal manipulation (166). Together, these approaches provide indirect indices of spinal-level processing that can be examined in relation to FBM-induced changes in afferent input and motor neuron excitability.

#### 3.4.2 D4.2 supraspinal and corticospinal assessment

Corticospinal and supraspinal processes can be assessed using non-invasive brain stimulation and neurophysiological recording techniques. Motor evoked potentials elicited with transcranial magnetic stimulation (TMS) provide measures of corticospinal excitability that reflect contributions from cortical, subcortical, spinal, and peripheral motor pathways. Spinal manipulation has been examined using TMS-based measures, including studies of central motor excitability after manipulation (169), cortical drive to upper and lower limb muscles following spinal manipulation (170), TMS-induced I-wave excitability and cortical silent period responses after chiropractic spinal manipulation (171), and changes in strength and cortical drive in athletes following a single spinal manipulation session (172). These studies illustrate how corticospinal assessment can be used to evaluate whether FBM is associated with changes in motor pathway excitability or motor output.

Electroencephalography (EEG) and magnetoencephalography provide high temporal resolution measures of cortical activity, enabling quantification of neural oscillations, event-related responses, and functional connectivity patterns (173–177). Somatosensory evoked potential studies have reported changes in sensorimotor integration following cervical spine manipulation (132, 178), while sLORETA-based EEG approaches have been used to examine central processing of tonic pain after chiropractic spinal manipulation (179). Resting-state EEG connectivity has also been explored following chiropractic spinal manipulation in neurological populations (177). These approaches are relevant for characterizing the supraspinal encoding of FBM-related somatosensory inputs and for examining whether FBM modulates cortical or network-level processing.

Functional MRI provides complementary spatial information about supraspinal responses associated with FBM. As described in Domain 2, fMRI studies have evaluated cerebral hemodynamic responses to pain following thoracic thrust manipulation, resting-state connectivity after manual therapy, Neurologic Pain Signature activation following thoracic spinal manipulation, and pain-related brain connectivity changes associated with manual therapy (180). Within this neurophysiological domain, these imaging studies are interpreted as measures of supraspinal processing rather than simply imaging outcomes. However, systematic reviews of brain-based responses to spinal manipulation emphasize that findings remain heterogeneous and should be interpreted cautiously with respect to mechanism and clinical meaning (181).

#### 3.4.3 D4.3 afferent encoding and microneurography

Mechanical inputs applied during FBM are likely to engage a range of mechanoreceptors, including muscle spindles, Golgi tendon organs, joint mechanoreceptors, and cutaneous receptors. Direct measurement of these afferent pathways in humans remains challenging, but animal studies using single-unit recordings provide important experimental evidence that manipulation-like mechanical inputs can alter primary afferent responsiveness. Studies of paraspinal muscle spindle afferents have shown that spinal manipulation parameters, including force magnitude, thrust duration, preload, and vertebral displacement, can influence spindle discharge and responsiveness (137, 182–186). These studies provide direct experimental support for the concept that specific FBM force-time characteristics can shape the sensory input entering spinal circuits.

Microneurography provides a method for recording single-unit afferent activity in peripheral nerves *in vivo*, enabling characterization of afferent discharge patterns, recruitment thresholds, adaptation behavior, and receptor classification. Although direct human microneurography studies of FBM remain limited, the method represents a powerful approach for future studies seeking to characterize how manual or instrument-assisted mechanical inputs are encoded by cutaneous, muscle, or joint afferents. C-tactile afferents, low-threshold unmyelinated mechanoreceptors that respond preferentially to gentle stroking touch, may be particularly relevant for lighter-touch or massage-like FBM approaches because of their links to affective touch, autonomic regulation, and pain modulation.

Together, neurophysiological monitoring methods help bridge externally applied mechanical inputs with afferent encoding, spinal excitability, motor output, supraspinal processing, and pain modulation. When integrated with biomechanical force measurement, imaging, sensory testing, vascular assessment, molecular assays, and clinical outcomes, these approaches can strengthen mechanistic inference about how FBM interacts with the nervous system.

### 3.5 Domain 5: vascular and microcirculation assessment

This domain includes methods for characterizing vascular, microcirculatory, lymphatic, and fluid-related responses to FBM (60, 187–189). These approaches quantify blood flow, perfusion, oxygenation, vascular tone, and tissue fluid dynamics across local and systemic levels. In FBM research, vascular and microcirculatory measures are relevant because applied mechanical forces may influence vessel compression, shear-related responses, lymphatic transport, autonomic regulation, and tissue metabolic demand. Compared with neurophysiological and sensory outcomes, vascular and lymphatic mechanisms remain less extensively studied in FBM; however, existing massage, manual therapy, and vascular imaging studies demonstrate feasible approaches for measuring circulatory and fluid-related responses.

#### 3.5.1 D5.1 blood flow and perfusion dynamics

Near-infrared spectroscopy (NIRS) and functional NIRS (fNIRS) quantify changes in tissue oxygenation through measurement of oxygenated and deoxygenated hemoglobin signals, reflecting aspects of local oxygen availability and hemodynamic response in muscle and cortical tissues (190). When applied in experimental settings, NIRS and fNIRS can be used to compare oxygenation patterns across conditions relevant to FBM and to characterize the temporal dynamics of perfusion changes following force application (191–193). In a recent chronic neck pain case study, fNIRS was used to assess cortical hemodynamic changes during a combined intervention that included manual therapy and pain neuroscience-based sensorimotor retraining, illustrating how hemodynamic monitoring can be incorporated into manual therapy research designs while also highlighting the need for more controlled FBM-specific studies (190).

Peripheral vascular function has also been measured directly in manual therapy contexts. Massage therapy has been shown to restore peripheral vascular function after exertion, providing a direct example of using vascular outcomes to assess manual therapy effects on circulatory physiology (194). Although massage is not identical to all forms of FBM, this study illustrates how vascular responsiveness can be used as a mechanistic outcome for interventions involving externally applied mechanical loading. Furthermore, characterizing FBM induced vascular responses such as those involving sustained pressure or compression, provides a targeted physiological model for these measurements. In this context, vascular imaging and doppler flowmetry serve as essential technologies to quantify how mechanical inputs safely regulate regional microvascular behavior, decoupling the therapeutic effect from pure mechanical restriction.

Laser Doppler flowmetry provides continuous measurement of superficial microvascular blood flow by assessing the flux of moving red blood cells within a sampled volume, yielding relative indices of microcirculatory perfusion that can be compared across experimental conditions (195). Laser speckle contrast imaging extends this capability to spatially resolved perfusion mapping, enabling assessment of regional microvascular responses to mechanical stimulation across tissue surfaces. These methods are well suited for testing whether manual or instrument-assisted loading produces local changes in skin or superficial soft-tissue blood flow, although direct FBM-specific laser Doppler and laser speckle studies remain limited.

Microvascular ultrasound techniques, including superb microvascular imaging (SMI), enable visualization of small-vessel flow patterns in deeper tissues, providing measures of flow characteristics and vascular distribution across tissue layers (196). Micro-computed tomography has been used to visualize blood vessel structure and angiogenesis in the vicinity of injured knee ligaments in response to instrument-assisted soft tissue manipulation (197). Plethysmography measures changes in limb volume or pressure, providing indirect indices of vascular compliance, venous return, and blood flow dynamics (198, 199). Photoplethysmography and capillaroscopy provide non-invasive measures of peripheral vascular function and capillary morphology, respectively, enabling assessment of microvascular structure and function at the tissue level. These approaches may be useful for evaluating how FBM influences local perfusion, vascular tone, or peripheral vascular reactivity, particularly when paired with measures of autonomic state from Domain 7.

Vascular imaging has also been used in cervical manipulation research to assess blood flow in safety- and physiology-relevant contexts. For example, vertebral artery blood flow has been measured across head positions and following cervical manipulative therapy, demonstrating how vascular flow assessment can inform interpretation of cervical FBM procedures and their vascular implications (200).

#### 3.5.2 D5.2 Lymphatic and fluid-related assessment

Lymphatic assessment methods provide measures of lymphatic vessel function and fluid transport that are relevant to FBM approaches targeting edema, tissue congestion, and lymphatic circulation. Near-infrared fluorescence lymphatic imaging using indocyanine green provides direct visualization of lymphatic vessel architecture and transport dynamics, enabling assessment of lymphatic propulsion, dermal backflow patterns, and fluid clearance. Although direct imaging studies of lymphatic responses to FBM remain limited, these methods are well positioned to evaluate manual lymphatic drainage, compression-based approaches, and other mechanically directed interventions intended to influence fluid transport.

Capillaroscopy enables direct visualization of capillary morphology and flow patterns at the nail fold or skin surface, providing measures of microvascular structural integrity and functional reserve. Infrared thermography provides spatial mapping of surface temperature, which may reflect vascular or autonomic processes associated with FBM, including changes in sympathetic vasoconstrictor tone and regional perfusion (201, 202). Bioimpedance spectroscopy provides non-invasive measures of tissue fluid distribution and extracellular water content, enabling assessment of FBM-related changes in tissue hydration, edema, and fluid redistribution.

Together, vascular, microcirculatory, lymphatic, and fluid-related methods provide tools for evaluating whether FBM influences local or systemic circulatory physiology. These measures are most informative when synchronized with mechanical input, autonomic state, tissue imaging, sensory testing, and clinical outcomes, allowing investigators to determine whether changes in blood flow, oxygenation, vascular tone, or fluid distribution accompany or mediate FBM-related responses.

### 3.6 Domain 6: computational modeling, data science, and finite element analysis (FEA)

This domain includes analytical and computational approaches for integrating multi-domain measurements into quantitative models of FBM mechanics and physiology. These methods are important because many mechanistically relevant variables, such as internal tissue stress, strain, fluid pressure, joint loading, and system-level response patterns, cannot be measured directly *in vivo*. Computational modeling and data science provide tools for estimating these internal states, testing mechanistic hypotheses, and identifying relationships across biomechanical, imaging, neurophysiological, vascular, molecular, sensory, contextual, and clinical data.

#### 3.6.1 D6.1 predictive biomechanical modeling

Musculoskeletal modeling uses kinematic, kinetic, and anatomical data to estimate joint moments, muscle forces, coordination strategies, and internal loading conditions during movement. These models can help determine how FBM-related changes in movement behavior or external loading may alter internal mechanical demands across joints, muscles, and soft tissues. Musculoskeletal modeling platforms, including OpenSim-based inverse kinematics and inverse dynamics workflows, provide a generalizable structure for estimating internal mechanical variables from externally measured motion and force data (203–208). In FBM research, these approaches are most useful when paired with direct measurement of applied force and motion, allowing investigators to estimate how externally applied mechanical inputs may alter internal loading or movement coordination.

Finite element analysis (FEA) and multi-body dynamics simulations extend this framework to tissue- and joint-level mechanics (209). FEA provides estimates of stress, strain, fluid-related behavior, and load distribution within tissues such as intervertebral discs, cartilage, ligaments, fascia, tendon, and muscle, based on subject-specific geometry, boundary conditions, and assumed material properties. Direct FBM applications of FEA are emerging (210). For example, FEA has been used to explore biomechanical mechanisms of cervical rotation manipulation across different bending positions, illustrating how modeling can estimate internal tissue loading patterns that cannot be measured directly during manipulation (211–214). Such models can help evaluate how variation in force direction, body position, contact location, or tissue properties may influence internal mechanical environments.

Subject-specific modeling also provides a way to translate imaging and motion data into estimates of tissue-level mechanics relevant to FBM planning and interpretation. For example, subject-specific estimates of cervical facet capsular ligament mechanics based on anatomy and kinematics demonstrate how imaging-derived geometry and motion data can be used to estimate soft tissue mechanical behavior in the spine (215). Although not a direct FBM intervention study, this type of modeling can inform FBM research by identifying tissue-level responses, constraints, or risk-relevant mechanical environments that may influence how externally applied forces are transmitted through spinal structures.

Image-based modeling integrates anatomical imaging with motion data to create subject-specific representations of structure and movement. CT, MRI, PETRA, or other imaging-derived geometries can generate individualized models, while high-fidelity motion data, such as biplane videoradiography from Domain 1, can provide experimentally measured bone motion for model inputs or validation. These approaches are particularly useful for linking external FBM delivery parameters to internal joint and tissue mechanics. Physics-informed neural networks and other hybrid modeling approaches provide an emerging strategy for combining physical laws with data-driven learning, enabling estimation of tissue mechanical behavior from sparse or incomplete experimental measurements.

#### 3.6.2 D6.2 data science, signal processing, and machine learning

Signal processing methods extract interpretable features from complex time-varying data. Examples include EMG decomposition, time-frequency analysis, wavelet transforms, independent component analysis, filtering, event detection, and synchronization of multimodal signals. These approaches are essential for aligning force delivery, movement, neurophysiological activity, autonomic signals, vascular responses, quantified contextual metrics, and patient-reported outcomes across time. For example, EMG decomposition and time-frequency methods can help characterize neuromuscular responses to FBM, while synchronization methods allow investigators to relate the timing of applied force to spinal reflex activity, cortical responses, vascular changes, or sensory outcomes.

Machine learning and data-driven approaches provide tools for analyzing high-dimensional, multimodal datasets. These methods can support automated image segmentation, pose estimation, physiological signal classification, response phenotyping, clustering, prediction, and statistical modeling of relationships among variables. Deep learning approaches have been applied broadly in musculoskeletal imaging for lesion detection, progression assessment, and prediction of musculoskeletal disease, while markerless pose-estimation tools such as DeepLabCut demonstrate how machine learning can extract movement features from video data (216–219). In FBM research, these tools may help scale measurement of movement behavior, tissue state, and physiological response across larger or more naturalistic datasets.

At present, machine learning applications using fully integrated, multi-domain FBM datasets remain limited. Therefore, data-driven models should generally be viewed as tools for feature extraction, response phenotyping, hypothesis generation, and prediction rather than as stand-alone evidence of mechanism. Conceptual modeling work in manual therapy has emphasized the need to link mechanical input, neurophysiological response, pain modulation, contextual factors, and clinical outcomes within integrated explanatory models (220). Computational and data science methods provide the analytical infrastructure needed to operationalize this goal, particularly when combined with experimental perturbation, well-controlled study designs, and standardized cross-domain data collection.

Together, computational modeling, data science, and FEA provide a bridge between measured external inputs and estimated internal mechanical or physiological states. These approaches can inform FBM procedures by estimating how force direction, dosage, posture, anatomy, and tissue properties may influence internal loading, and they can support FBM effect measurement by integrating mechanical, neural, vascular, molecular, sensory, contextual, and clinical data into testable mechanistic models.

### 3.7 Domain 7: sensory, psychophysical, and contextual assessment

This domain includes methods for characterizing the individual sensory, psychological, autonomic, and contextual factors that may shape responses to FBM. These approaches are important because the same externally applied mechanical input may produce different responses depending on pain sensitivity, endogenous pain modulation, autonomic state, expectations, prior experience, psychological factors, and the broader clinical context (221–223). By quantifying these factors, sensory and contextual assessments help explain heterogeneity in FBM response and support integration of biological mechanisms with patient-centered outcomes. Importantly these contextual variables function as dynamic feedback mechanisms. Future frameworks should account for how the patient and clinical environment interact to shape the mechanical dose applied by the clinician and subsequent physiological responses.

#### 3.7.1 D7.1 sensory thresholds, pain modulation, and descending inhibitory control

Standardized Quantitative Sensory Testing (QST) protocols provide sensory profiles that may include thermal detection and pain thresholds, mechanical detection thresholds, vibration detection thresholds, pressure pain thresholds, and wind-up or temporal summation of pain (224–227). These measures characterize sensory profiles that may reflect peripheral sensitization, central sensitization, or altered nociceptive processing. Pressure algometry is particularly relevant to FBM research because it provides localized measures of mechanical pain sensitivity that can be assessed before and after intervention (228–231).

QST and related psychophysical measures have been used directly to evaluate FBM-associated effects on pain sensitivity and hypoalgesia. A systematic review and meta-analysis reported changes in pain sensitivity following spinal manipulation, supporting the use of sensory testing as a mechanistic outcome in SMT research (232). Experimental studies have also shown spinal manipulative therapy-specific changes in pain sensitivity in individuals with low back pain (233), while other work has examined whether back pain relief after spinal manipulation is associated with decreased temporal summation of pain (234). Comparisons of lumbar manipulation and mobilization have further used temporal summation paradigms to distinguish immediate psychophysical effects across manual therapy approaches (235). Together, these studies illustrate how sensory testing can move beyond symptom reporting to evaluate candidate pain-modulatory mechanisms of FBM.

Pain modulation paradigms provide additional information about endogenous pain regulatory systems. Conditioned pain modulation (CPM) assesses the capacity of descending inhibitory pathways to suppress nociceptive input, while temporal summation of pain evaluates facilitation of pain responses to repeated stimuli (236). These approaches can help determine whether FBM is associated with changes in pain inhibition, pain facilitation, or sensory processing state. Other paradigms, such as offset analgesia or diffuse noxious inhibitory control protocols, may provide complementary measures of endogenous pain regulation depending on the study population and mechanistic question.

#### 3.7.2 D7.2 autonomic nervous system reactivity

Autonomic and psychophysiological measures provide indices of arousal and regulatory state that may influence or reflect responses to FBM. Heart rate variability, electrodermal activity, respiration, blood pressure, skin temperature, and pupillometry are commonly used to assess sympathetic and parasympathetic activity, autonomic flexibility, and physiological arousal (237–242). These measures may be particularly relevant when evaluating FBM approaches hypothesized to influence relaxation, stress physiology, pain modulation, or autonomic regulation.

Manual therapy and spinal manipulation studies have used autonomic outcomes to evaluate whether FBM alters physiological regulation, but the evidence remains mixed and often limited by methodological heterogeneity and low certainty. Systematic reviews have examined the effects of spinal manipulation, joint manipulative techniques, and manual therapy on autonomic nervous system markers such as heart rate variability, electrodermal activity, blood pressure, and related physiological outcomes (243, 244). More recent overviews emphasize that manual therapy may influence sympathetic and parasympathetic measures, but findings are heterogeneous and depend on technique, region treated, population, outcome measure, and methodological quality (245, 246).

Autonomic measures can be synchronized with force delivery, sensory testing, neurophysiological monitoring, or clinical outcomes to examine temporal relationships between mechanical input and physiological response. For example, changes in electrodermal activity, heart rate variability, skin temperature, or pupillometry during or after FBM may provide information about arousal, threat processing, affective state, or regulatory response. These measures do not identify a mechanism in isolation, but they can strengthen interpretation when integrated with mechanical, neural, sensory, vascular, and patient-reported outcomes.

#### 3.7.3 D7.3 psychometric and ecological momentary assessment

Psychometric instruments and patient-reported outcome measures characterize the subjective and contextual dimensions of FBM response. Measures of pain intensity, function, quality of life, treatment expectations, pain catastrophizing, fear avoidance, self-efficacy, anxiety, depression, sleep, and perceived improvement provide information about the patient-level context in which FBM is delivered and experienced. Common examples include pain intensity ratings or the PEG scale, Oswestry Disability Index, Neck Disability Index, Patient-Reported Outcomes Measurement Information System measures, Pain Catastrophizing Scale, Pain Self-Efficacy Questionnaire, Fear-Avoidance Beliefs Questionnaire, Patient Health Questionnaire-9, Generalized Anxiety Disorder-7, Credibility/Expectancy Questionnaire, and Patient Global Impression of Change. These variables may act as moderators, mediators, or outcomes depending on the study design. Beyond patient psychometrics, standardizing the measurement of the external environment and provider-patient interaction is critical for isolating the mechanical signal. Treating the physical and relational environment as a quantified metric, rather than unmeasured variance, enables investigators to control for the contextual modifiers of the mechanical input.

Contextual and expectation-related measures are particularly important in FBM research because treatment response may depend not only on the mechanical input but also on patient expectations, therapeutic context, provider interaction, and prior experience. Experimental and clinical studies have shown that expectation can influence spinal manipulation-induced hypoalgesia and clinical outcomes following manual therapy (222, 247–254). These studies demonstrate that psychometric and contextual measures are not simply ancillary outcomes; they can help explain why similar FBM inputs produce different responses across individuals.

Ecological momentary assessment (EMA) extends these measurements into real-world settings by capturing repeated, time-stamped assessments of pain, mood, activity, sleep, medication use, and perceived treatment response in daily life. EMA and related diary-based approaches can help characterize the temporal dynamics of FBM response outside the laboratory, including variability across days, activities, and contexts. When paired with wearable sensors, ambulatory biomechanical monitoring, or repeated clinical outcomes, EMA can link subjective symptoms with movement behavior, activity exposure, and real-world functional change. Although EMA is not yet widely established in FBM mechanistic studies, it provides a practical approach for connecting short-term laboratory responses to patient-centered outcomes in daily life.

Together, sensory, psychophysical, autonomic, and contextual assessments help explain why FBM responses vary across individuals and settings. When integrated with biomechanical, imaging, neurophysiological, vascular, molecular, and computational approaches, these methods provide the patient-centered and context-sensitive data needed to interpret mechanistic findings in relation to clinically meaningful outcomes.

### 3.8 Domain 8: mechanistic perturbation and neuromodulation methods

This domain includes approaches used to experimentally perturb specific components of the sensory, neuromuscular, vascular, or central nervous systems. Unlike the preceding domains, which primarily measure aspects of FBM input or response, these methods provide mechanistic leverage by altering a targeted system component and observing whether the FBM response changes (255). When combined with measurement approaches from Domains 1–7, perturbation and neuromodulation methods can strengthen causal inference by helping determine whether a specific pathway is necessary, sufficient, or modulatory for a given FBM-associated response.

#### 3.8.1 D8.1 biofeedback, virtual/augmented reality, and mechanical perturbation paradigms

Biofeedback systems provide a method for modifying sensory input, motor output, or treatment delivery in a controlled manner (48, 256, 257). Visual, auditory, and haptic feedback can be used to alter movement patterns, muscle activation, postural control, or force application. In FBM research, real-time force feedback is particularly valuable because it allows providers to monitor and standardize parameters such as force magnitude, rate, contact location, direction, and duration. Quantifiable soft tissue manipulation systems provide a direct example of this approach, using instrumented localizing and dispersive devices to capture real-time force-motion patterns during manual therapy delivery (23, 24). In this context, feedback systems serve not only as measurement tools, but also as experimental controls that allow investigators to standardize or systematically vary FBM input parameters.

Virtual reality and augmented reality systems allow controlled manipulation of sensory and contextual inputs. These systems can modify visual, proprioceptive, vestibular, attentional, and affective components of the treatment environment, making them useful for examining how perception, expectation, threat, body representation, or sensory context influences responses to mechanical input. Although direct VR/AR applications during FBM remain limited, these technologies provide a practical strategy for experimentally manipulating contextual state while measuring mechanical, sensory, neurophysiological, or clinical outcomes. In future FBM studies, VR/AR could help separate the contribution of mechanical input from expectation, attention, and environmental context.

Mechanical perturbation and vibration paradigms provide controlled ways to activate sensory and neuromuscular pathways (129, 258–260). Local vibration, tendon vibration, stochastic resonance approaches, and whole-body vibration can preferentially engage muscle spindle afferents, cutaneous receptors, or other mechanosensitive pathways depending on the stimulation parameters. These paradigms are relevant to FBM because they allow investigators to experimentally vary afferent input without reproducing a full manual therapy intervention. For example, vibration or mechanically induced perturbations can be paired with EMG, H-reflex, postural control, or sensory testing outcomes to determine how specific classes of mechanoreceptor input influence reflex behavior, motor output, or pain modulation. These approaches should be considered FBM-relevant mechanistic analogs rather than direct substitutes for clinical FBM.

#### 3.8.2 D8.2 pharmacologic modulation and non-invasive brain stimulation

Pharmacologic modulation and nerve blocks provide approaches for altering neural, inflammatory, vascular, or biochemical pathways (261–263). Local anesthetic blocks can reduce afferent input from targeted tissues, while pharmacologic agents may influence neurotransmitter systems, inflammatory signaling, vascular tone, muscle activation, or pain processing. In FBM research, these methods could be used to test whether peripheral afferent input, local inflammatory signaling, or specific neural pathways are necessary for an observed response to mechanical loading. However, direct pharmacologic-block studies designed specifically to isolate FBM mechanisms remain limited. Therefore, these approaches are best framed as important future mechanistic probes that require careful experimental design, safety oversight, and appropriate control conditions.

Non-invasive brain stimulation, including transcranial magnetic stimulation and transcranial electrical stimulation, provides tools for assessing or modulating cortical excitability and network activity. TMS-derived motor evoked potentials have already been used in spinal manipulation research to assess corticospinal excitability, cortical drive, I-wave excitability, and cortical silent period responses following manipulation (168, 170–172). Within Domain 4, these approaches function primarily as measurement tools. Within Domain 8, their value is as mechanistic probes: repetitive TMS or transcranial electrical stimulation approaches could be used to experimentally alter supraspinal excitability or network state before FBM to determine whether cortical excitability, sensorimotor integration, or pain-related network activity contributes to observed responses.

Because perturbation and neuromodulation methods actively alter physiological systems, their interpretation depends on specificity of the perturbation, appropriate sham or control conditions, temporal alignment with FBM delivery, and careful measurement of downstream outcomes (264). Their use also requires attention to safety, consent, and regulatory oversight. When applied within rigorous experimental designs and combined with measures from other domains, these approaches can help move FBM research beyond association toward stronger tests of mechanism.

Together, mechanistic perturbation and neuromodulation methods provide experimental leverage for testing candidate pathways. By standardizing or manipulating force delivery, altering sensory context, modifying afferent input, blocking peripheral pathways, or modulating supraspinal excitability, these approaches can help determine which components of the FBM response are mechanically driven, contextually mediated, neurally regulated, or biologically downstream of the applied force.

## 4 Discussion

### 4.1 Overcoming challenges of multi-domain study design

The methodological landscape for investigating FBM spans multiple domains and scales of biological organization. The primary contribution of this review is not the introduction of new methodologies, but the structured integration of established approaches within a framework that enables coordinated, multi-domain investigation of relationships between mechanical input and measurable physiological responses.

A key insight emerging from this synthesis is that no single modality is sufficient to support mechanistic inference in FBM. Meaningful investigation requires coordinated integration across domains that collectively span the pathway from externally applied force to biological response while accounting for individual and contextual state. Domain 1 characterize applied mechanical inputs, movement behavior, joint motion, tissue displacement, and loading patterns. Domains 2–5 characterize tissue, cellular, neural, vascular, and physiological responses. Domain 6 provides computational and analytical approaches for integrating measurements and estimating internal states that cannot be measured directly. Domain 7 captures sensory, psychophysical, autonomic, psychological, and contextual factors that shape individual response. Domain 8 provides experimental leverage to perturb specific pathways and strengthen causal inference.

This framework has direct implications for study design. Studies should be structured to capture complementary points along the pathway from mechanical input to measured physiological response, rather than relying on single-domain outcomes alone. For example, a multi-domain study could combine measurement of applied forces during FBM, joint-level kinematics and tissue strain, spinal reflex excitability and cortical responses, pressure pain thresholds and conditioned pain modulation, and computational modeling of internal tissue mechanics within a single experimental paradigm. Such designs enable more comprehensive characterization of system-level responses than single-domain approaches can achieve.

Each methodological domain carries inherent limitations related to measurement specificity, spatial and temporal resolution, and assumptions underlying interpretation. Imaging-based approaches often reflect indirect proxies of underlying biological processes; neurophysiological measures capture system-level activity rather than isolated pathways; biomarker and molecular assays may identify biological associations without defining the mechanical origin of those responses; and computational models depend on assumptions regarding structure, material properties, boundary conditions, and input data. These limitations underscore the importance of integrating complementary methods and interpreting findings within the context of study design and measurement constraints.

Practical barriers, including the need for interdisciplinary expertise, challenges in synchronizing multi-modal datasets, variability in experimental protocols, and limited access to advanced instrumentation, continue to limit progress. Multi-domain studies require substantial investment in specialized equipment, personnel with diverse expertise, and longitudinal or multi-modal data collection. In this context, this review provides a shared organizational structure that can guide collaborative team science, enabling researchers with different domain expertise to contribute complementary measurements within integrated study designs. Practical recommendations for applying this framework in future FBM study design are summarized in Table 1.

**Table 1:** Design implications for future studies of force-based manipulation mechanisms.

Design implication	Application for future FBM studies	Methodological examples and supporting evidence
Integrate complementary domains	Combine measures that capture distinct but connected components of the proposed pathway from mechanical input to biological and clinical response.	Applied-force measurement may be combined with segmental motion, tissue deformation, EMG or reflex measures, neuroimaging, sensory testing, clinical outcomes, and computational modeling. For example, instrumented manipulation has been related to paraspinal muscle responses, while BVR has directly linked manipulation to vertebral motion (38, 148).
Characterize the mechanical input explicitly	Define the FBM exposure using sufficient mechanical and procedural detail to support replication and comparison across studies.	Relevant parameters include force magnitude, direction, rate, duration, contact area, dosage, body region, delivery method, and provider or device characteristics. Instrumented FBM and QSTM systems can capture objective force–motion patterns, improving reproducibility, dose characterization, and comparisons across techniques (23, 24).
Align outcomes with a proposed mechanistic pathway	Select outcomes that directly measure the components and sequence specified in the study's mechanistic hypothesis.	A neurophysiological study might combine force–time characteristics with segmental motion, afferent recordings, EMG, H-reflex testing, sensory measures, and clinical outcomes. Experimental studies have related manipulation parameters to muscle-spindle afferent activity and assessed spinal excitability following manipulation (137, 166, 183, 186).
Match measurement timing to the expected response	Schedule measurements according to the anticipated onset and duration of the mechanical, physiological, or biological response.	Tissue deformation, afferent encoding, muscle activation, and reflex responses may occur during or immediately after FBM. Autonomic, vascular, and sensory responses may emerge over minutes, whereas inflammatory, metabolic, remodeling, behavioral, and clinical responses may require hours, days, or longer follow-up (93, 126, 148).
Account for individual and contextual modifiers	Measure participant, provider, and environmental factors that could modify the relationship between the applied input and observed response.	Relevant variables include baseline tissue state, pathology, pain chronicity, sensitization, autonomic state, expectations, psychological factors, prior treatment exposure, and provider–patient context. Psychometric assessment, autonomic monitoring, ecological momentary assessment, and patient-reported outcomes can help determine why similar mechanical inputs produce different responses (222, 247, 248).
Distinguish direct FBM evidence from analogs and emerging tools	State whether each method or finding represents direct FBM evidence, a mechanistic analog, or a future-facing approach.	Direct evidence includes instrumented FBM delivery, *in vivo* motion measurement, neurophysiological testing, imaging, and quantitative sensory assessment. Animal and modeled manual-therapy studies provide mechanistic analogs for inflammation, fibrosis, nociceptor activity, and tissue remodeling, whereas VR/AR, pharmacologic modulation, and some neuromodulation methods remain emerging causal probes (38, 93, 123).
Build interdisciplinary study teams	Include the methodological and clinical expertise required to acquire, integrate, and appropriately interpret measurements across the selected domains.	Multi-domain studies may require expertise in biomechanics, imaging, neurophysiology, vascular or molecular biology, sensory assessment, data science, clinical research, and implementation science. The NCCIH FBM Research Networks provide a model for coordinated investigation across institutions and disciplines (1, 5).
Design studies for scalability and data integration	Use standardized measures, terminology, and data structures that can support pooled analyses, multi-site studies, and clinical registries.	Standardized force reporting, shared terminology, harmonized outcome batteries, validated contextual measures, and interoperable data structures are needed to integrate mechanical, biological, physiological, contextual, and clinical data across studies and registries (265).

Future FBM studies should be designed to link measured mechanical inputs with complementary biological, physiological, contextual, and clinical outcomes. This requires explicit characterization of force delivery, careful alignment of outcomes with hypothesized mechanisms and time scales, interdisciplinary collaboration, and scalable data infrastructure that supports integration across sites and domains.

### 4.2 Interpreting direct FBM evidence, mechanistic analogs, and emerging tools

The examples summarized across domains show that FBM research already has several areas of direct mechanistic evidence. Instrumented force measurement and quantifiable soft tissue manipulation systems demonstrate that applied manual inputs can be objectively characterized. Biplane videoradiography and related motion analysis approaches demonstrate that spinal manipulation and clinically relevant movement patterns can be linked to segment-level motion. Ultrasound elastography, fMRI, PET, and quantitative MRI demonstrate that imaging can measure tissue, metabolic, or central nervous system variables relevant to FBM. Animal and modeled manual therapy studies provide evidence that mechanical loading can influence inflammation, fibrosis, nociceptor activity, neural fibrosis, and tissue remodeling. Neurophysiological studies using EMG, H-reflex, TMS, EEG, fMRI, and animal afferent recordings demonstrate that FBM-related inputs can be studied across peripheral, spinal, and supraspinal levels. Quantitative sensory testing and psychophysical approaches demonstrate that spinal manipulation and manual therapy can be evaluated in relation to pain sensitivity, temporal summation, expectation, and other response-modifying factors.

At the same time, not all technologies are equally established within FBM research. Some methods have been used directly to measure FBM effects, whereas others are better described as FBM-relevant tools or mechanistic analogs (232, 233, 248). For example, vascular and lymphatic methods provide plausible and feasible approaches for studying fluid-related responses, but the direct evidence base is thinner than in neurophysiology or sensory testing. Virtual reality, augmented reality, pharmacologic block, and some neuromodulatory approaches may be valuable causal probes, but they should not yet be treated as established FBM mechanism measures.

This distinction is important. The goal of this review is not to imply that all listed technologies have already been fully validated in FBM contexts. Rather, it is to clarify where methods have already provided mechanistic insight, where adjacent mechanobiology or rehabilitation research provides a strong rationale, and where emerging tools could help address unresolved questions. Explicitly distinguishing direct FBM evidence from mechanistic analogs and future-facing methods helps prevent overinterpretation while still identifying opportunities for study design.

### 4.3 Timing, context, and interpretation of multi-domain responses

A recurring challenge in FBM research is that responses may occur over different time scales. Some effects occur within milliseconds during force application, including tissue deformation, afferent encoding, and neuromuscular activation. Other responses may emerge over seconds to minutes, including spinal reflex modulation, autonomic shifts, perfusion changes, or altered pain sensitivity. Still others may unfold over hours, days, or weeks, including inflammatory, metabolic, molecular, tissue remodeling, behavioral, and clinical responses. A method that is highly informative at one time scale may be poorly suited to another.

Context and population heterogeneity further shape interpretation. The same applied mechanical input may produce different responses depending on tissue state, pain chronicity, sensitization, expectations, psychological state, autonomic arousal, prior treatment exposure, age, sex, pathology, provider-patient interaction, and the physical clinical environment. This is why sensory, psychophysical, autonomic, and contextual assessments are not secondary to mechanistic FBM research. They are essential for understanding why responses vary across individuals and why a mechanical input may produce different outcomes in different biological or clinical contexts.

Integrated study designs should therefore define not only what was applied, but also when outcomes were measured, in whom they were measured, and under what contextual conditions. Studies that combine force measurement with biological, physiological, sensory, contextual, and clinical outcomes will be better positioned to evaluate candidate mechanisms than studies relying on single-domain outcomes alone.

### 4.4 Scaling the lab to clinical registries

The long-term goal of FBM mechanistic research is translation: linking laboratory-derived mechanistic insights to clinically meaningful outcomes and ultimately to optimized, personalized FBM practice. Achieving this requires infrastructure that supports coordinated data collection across sites, standardized reporting of variables, and integration of mechanistic measurements with clinical outcome registries.

Scaling from laboratory to clinical registries will require several developments: standardized force parameter reporting protocols that enable comparison of intervention variables across studies and clinical settings; harmonized outcome measure batteries that capture biological responses at multiple levels; validated contextual factor assessments and consolidated instrument toolkits that characterize the psychosocial and environmental context of FBM delivery; and data infrastructure that supports multi-site, multi-modal data integration and sharing.

Standardized force reporting is especially important. Studies should describe applied mechanical inputs using parameters such as magnitude, direction, rate, duration, contact area, dosage, delivery method, body region, and provider or device characteristics whenever possible. These descriptions are necessary for comparing findings across studies and for relating external inputs to internal tissue, neural, vascular, molecular, sensory, and clinical responses. This approach aligns with broader interprofessional efforts to improve manual therapy nomenclature by emphasizing descriptive mechanical parameters and shared terminology rather than relying solely on profession-specific labels (265).

The NCCIH Force-Based Manipulations Research Networks, including SPINEWORK, ForceNET, and NeuronS_MATTR, provide a critical foundation for this infrastructure, supporting coordinated, multi-domain research efforts across institutions and disciplines. By organizing measurement technologies into shared methodological domains, this review provides common language needed to aggregate, compare, and interpret findings across studies that sample different regions of the FBM response pathway.

## 5 Conclusion

FBM are widely recommended and commonly used clinical interventions in pain management, rehabilitation, wellness, and musculoskeletal care, yet the biologic processes associated with their effects remain incompletely characterized. This review demonstrates that the methodological tools required to investigate these processes are well established and span a comprehensive range of physiologic systems and scales. The central challenge is not simply identifying new tools, but organizing existing and emerging methodologies within a shared framework that links externally applied mechanical FBM inputs to measurable biological responses and clinically meaningful outcomes.

The eight domains reviewed here collectively cover applied force measurement and movement analysis; tissue, cellular, and molecular imaging; biomarker, transcriptomic, and mechanobiological analysis; neurophysiological monitoring; vascular and fluid-related physiology; computational modeling, data science, and finite element analysis; sensory, psychophysical, and contextual assessment; and mechanistic perturbation and neuromodulation.

Advancing the science of FBM will require coordinated, multi-domain study designs that integrate measurements across scales and systems and link them to clinically meaningful outcomes. This will depend on standardization of force parameter reporting, harmonization of biological and clinical outcome measures, systematic characterization of physical and relational contextual factors, and infrastructure that supports interdisciplinary collaboration and cross-site data integration.

Together, the FBM Research Networks and the growing community of interdisciplinary FBM researchers will continue to make strides in the development of rigorous, integrative studies that advance understanding of how FBM relate to physiological processes and clinical outcomes and, ultimately, how they can be optimized to benefit patients.

## In Memoriam

This work is dedicated to the memory of Dr. Greg N. Kawchuk, whose scientific insight, mentorship, and leadership were instrumental in shaping this work and the broader field of force-based manipulation research.
