# Supplementary material for: Technologies to investigate the mechanisms of force-based manipulations: a narrative methods review
Source: Front Pain Res (Lausanne). 2026 Aug 28;7:1908955. doi: 10.3389/fpain.2026.1908955 (PMC13563129; doi:10.3389/fpain.2026.1908955)
Supplement: Supplementary file 1 [file Datasheet1.zip › FBM_Hierarchical_Taxonomy_with_citations.html]

Supplementary Material. Hierarchical Taxonomy of Methodologies and Technologies for FBM Mechanism Research


# Supplementary Material. Hierarchical Taxonomy of Methodologies and Technologies for FBM Mechanism Research

Expandable domain-by-domain outline of method families used to investigate mechanisms of force-based manipulation (FBM).


Expand all
Collapse all
Print / Save PDF

Domain 1
Domain 2
Domain 3
Domain 4
Domain 5
Domain 6
Domain 7
Domain 8

Use the arrows to expand or collapse each domain and subsection. The search box opens matching sections automatically and highlights matching terms. Domain colors are matched to Figure 1. Representative citations from the main manuscript are included within each subsection; full reference details are provided in the main manuscript.

Domain 1. Biomechanical Force and Motion Analysis

characterizes applied mechanical inputs, movement behavior, joint motion, tissue displacement, and loading patterns.

D1.1 Applied Force Quantification

- Force plates and instrumented treadmills
- Dynamometry, including handheld, isokinetic, grip-based, and task-specific systems
- Arthrometry
- Quantifiable soft tissue manipulation (QSTM) systems
- Robotic and haptic systems
- Shear and torsional force platforms

Representative citations: (Barbe, Panibatla, et al., 2021; Collings et al., 2024; Collins et al., 2009; DeBerardinis et al., 2018; Batista et al., 2024; Chamorro et al., 2017; Landsman et al., 2023, 2026; Núñez-Cortés et al., 2022; Schindler et al., 2023; Wiertsema et al., 2008; Bhattacharjee A et al., 2021; Bhattacharjee et al., 2023; Rodríguez-Fernández et al., 2021).


D1.2 In Vivo Motion Analysis

- Optical motion capture systems
- Biplane videoradiography (BVR)
- Markerless motion tracking approaches
- Inertial measurement units (IMUs)
- Electromagnetic tracking systems

Representative citations: (Carpino et al., 2020; Derian et al., 2020; Moreno et al., 2018; Romero-Flores et al., 2024; Svoboda et al., 2025; Ellingson et al., 2013; Kage et al., 2020, 2026; Knörlein et al., 2016; Morton et al., 2025; Anderst, 2015; Anderst et al., 2015, 2018; Lam et al., 2023; Ray et al., 2024; Varcin & Boocock, 2026; Wade et al., 2022; He et al., 2024; S. Lin et al., 2025; Mundt et al., 2019).


D1.3 Tissue Deformation

- Indentation testing, compliance testing, and digital image correlation (DIC)
- Skin and soft-tissue deformation modeling
- Strain-based biomarkers
- Haptic interfaces

Representative citations: (Kao AR et al., 2025; Raghupathy & Barocas, 2010; Giggins et al., n.d.; Pacheco-Barrios et al., 2024).


D1.4 Ambulatory Monitoring and Wearable Kinematics

- Wearable sensor systems
- IMU-based systems
- Continuous monitoring of movement patterns, postural behavior, and physical activity in free-living conditions
- Patient-reported outcomes, ecological momentary assessment, and clinical performance measures

Representative citations: (He et al., 2024; S. Lin et al., 2025; Mundt et al., 2019).


Domain 2. Tissue, Cellular, and Molecular Imaging

quantifies structural, compositional, and functional characteristics of tissues across in vivo and ex vivo scales.

D2.1 Ultrasound Imaging and Elastography

- B-mode ultrasound
- Speckle-tracking ultrasound
- Ultrasound elastography, including strain and shear-wave elastography
- Doppler ultrasound methods, including color, power, and spectral Doppler
- Contrast-enhanced ultrasound (CEUS) and vector flow imaging

Representative citations: (Frich et al., 2019; Chowdhary et al., 2024; Ryu & Jeong, 2017; Taljanovic et al., 2017; Luomala et al., 2014; Devantéry et al., 2023; Ema et al., 2025; Olchowy et al., 2020; Jones et al., 2023; Paeng et al., 2025).


D2.2 Advanced MRI Techniques

- Conventional T1- and T2-weighted sequences
- Quantitative MRI techniques
- T1 and T2 mapping
- T1ρ
- Ultrashort echo time (UTE)
- PETRA
- dGEMRIC where applicable
- Dixon-based fat fraction imaging
- Diffusion tensor imaging (DTI) and diffusion spectrum imaging (DSI)
- Magnetic resonance elastography (MRE)
- Functional MRI (fMRI; BOLD)
- Perfusion MRI, including arterial spin labeling (ASL)

Representative citations: (Nikpasand et al., 2024; Afsahi et al., 2022; Bipin Mehta et al., 2019; De Mello et al., 2019; Ellingson et al., 2014; Kanal et al., 2025; Chianca et al., 2017, 2023; Dresner et al., 2001; Reiter et al., 2025; Schmidt et al., 2025; Du et al., 2024; Tan et al., 2020; Weber II et al., 2019; Wen et al., 2022; Sparks et al., 2013; Gay et al., 2014; Ellingsen et al., 2018, 2023).


D2.3 Radiographic Imaging

- Projection radiography
- Fluoroscopy
- Computed tomography (CT) and cone-beam CT (CBCT)
- EOS imaging
- Dual-energy X-ray absorptiometry (DXA)
- Single-photon emission computed tomography (SPECT)
- Positron emission tomography (PET)

Representative citations: (du Rose et al., 2021; Ibad et al., 2023; Chaudhari et al., 2021; Israel et al., 2019; Kogan et al., 2018; Ogura et al., 2011; Inami et al., 2017).


D2.4 Histologic and Microscopic Imaging

- Histology
- Immunohistochemistry
- Immunofluorescence
- Confocal microscopy
- Multiphoton microscopy
- Electron microscopy

Representative citations: (Kim et al., 2016; Loghmani & Warden, 2009; Magaki et al., 2019; Barbe, Harris, et al., 2021; Butterfield et al., 2008; Saitou et al., 2018; Nim et al., 2020).


Domain 3. Biomarker, Transcriptomic, and Mechanobiological Analysis

assesses biological signaling, mechanotransduction, inflammation, metabolism, and tissue adaptation.

D3.1 Systemic and Tissue Biochemical Marker Assays

- Enzyme-linked immunosorbent assays (ELISA)
- Western blotting
- Multiplex immunoassays
- Protein-based methods
- Microdialysis
- Serum, plasma, saliva, or tissue biomarker panels

Representative citations: (Anloague et al., 2020; Butler et al., 2019; Carson et al., 2015; Eaton et al., 2014; Loghmani et al., 2021; Zubair et al., 2026; Barbe, Panibatla, et al., 2021; Rosker & Sarabon, 2010; Bove et al., 2016; Mustaklem et al., 2025; Maggs et al., 1997; Olausson et al., 2013; Kovanur Sampath et al., 2024; Puerto Valencia et al., 2024; Rossettini et al., 2018).


D3.2 Transcriptomic, Proteomic, Metabolomic, and Epigenetic Responses

- Quantitative PCR
- Bulk RNA sequencing
- Single-cell RNA sequencing
- Proteomic profiling
- Metabolomic profiling
- Epigenetic analyses

Representative citations: (Conesa et al., 2016; de Jonge et al., 2022; Keane et al., 2024; Marques et al., 2011).


D3.3 Mechanobiological Signaling and Cellular Response Assays

- Mechanosensitive ion channels
- Piezo1, Piezo2, and TRPV4
- Mechanosensitive transcriptional regulators
- YAP, TAZ, and focal adhesion kinase (FAK)
- Modeled manual therapy studies
- Massage-like loading studies
- Histology, immunohistochemistry, immunofluorescence, and electron microscopy

Representative citations: (Cost et al., 2019; Berrueta et al., 2016; Bouffard et al., 2008; Hettinger et al., 2021; Thompson et al., 2016; Bove et al., 2016, 2017, 2019; Barbe, Panibatla, et al., 2021; Rosker & Sarabon, 2010; Andrzejewski et al., 2014, 2015; C. Haas, Butterfield, Abshire, et al., 2013; C. Haas, Butterfield, Zhao, et al., 2013; C. Haas et al., 2012; Butterfield et al., 2008; Miller et al., 2018).


Domain 4. Neurophysiological Monitoring

evaluates how mechanical inputs are encoded and processed across peripheral, spinal, and supraspinal nervous system pathways.

D4.1 Peripheral and Spinal Excitability

- Electromyography (EMG)
- Surface EMG
- High-density surface EMG
- Intramuscular EMG
- Mechanomyography
- Tensiomyography
- Nerve conduction studies
- Teased nerve fiber electrophysiology
- H-reflex and F-wave methodologies

Representative citations: (Bellosta-López et al., 2023; Hermens et al., 2000; Merletti & Muceli, 2019; Muceli & Merletti, 2024; Nawab et al., 2010; Niazi et al., 2020; Reaz et al., 2006; Taylor & Murphy, 2008; Nougarou F et al., 2013; Currie et al., 2023; Pagé I et al., 2018; Ibitoye et al., 2014; Islam et al., 2013; Kalc et al., 2023; Lohr et al., 2019; Burke et al., 2007; Ramani et al., 2026; Schmelz & Schmidt, 2010; Tamarkin & Isaacson, 2026; Bove et al., 2019; Moraska et al., 2008; Pratelli et al., 2015; Goldsworthy et al., 2016; Grosprêtre & Martin, 2012; Niazi et al., 2015; Suter et al., 2005; Dishman & Burke, 2003).


D4.2 Supraspinal and Corticospinal Assessment

- Motor evoked potentials
- Transcranial magnetic stimulation (TMS)
- Electroencephalography (EEG)
- Magnetoencephalography
- Somatosensory evoked potential studies
- sLORETA-based EEG approaches
- Resting-state EEG connectivity
- Functional MRI

Representative citations: (Dishman et al., 2002; Haavik et al., 2016, 2018, 2024; Christiansen et al., 2018; Beniczky & Schomer, 2020; Chaddad et al., 2023; Singh, 2014; Steven Waterstone et al., 2020; Haavik Taylor & Murphy, 2010; Haavik-Taylor & Murphy, 2007; Navid et al., 2019; L. Cao et al., 2026; Meyer et al., 2019).


D4.3 Afferent Encoding and Microneurography

- Animal studies using single-unit recordings
- Paraspinal muscle spindle afferents
- Microneurography
- C-tactile afferents

Representative citations: (Cao DY et al., 2013; Lima CR et al., 2020; Reed WR, Cao DY, et al., 2015; Reed WR et al., 2013, 2014; Reed WR, Pickar JG, et al., 2015).


Domain 5. Vascular and Microcirculation Assessment

measures blood flow, perfusion, oxygenation, lymphatic function, and fluid-related physiological responses.

D5.1 Blood Flow and Perfusion Dynamics

- Near-infrared spectroscopy (NIRS) and functional NIRS (fNIRS)
- Peripheral vascular function
- Laser Doppler flowmetry
- Laser speckle contrast imaging
- Microvascular ultrasound techniques
- Superb microvascular imaging (SMI)
- Plethysmography
- Photoplethysmography
- Capillaroscopy
- Vertebral artery blood flow

Representative citations: (Bae et al., 2025; Murkin & Arango, 2009; Tandirerung et al., 2024; Tuesta et al., 2022; Franklin et al., 2014; Eun, 1995; Aziz et al., 2022; Loghmani & Warden, 2013; Allen, 2007; Wilkinson & Webb, 2001; Yelverton et al., 2020).


D5.2 Lymphatic and Fluid-Related Assessment

- Near-infrared fluorescence lymphatic imaging
- Indocyanine green
- Capillaroscopy
- Infrared thermography
- Bioimpedance spectroscopy

Representative citations: (dos Santos Bunn et al., 2020; X. Li et al., 2020).


Domain 6. Computational Modeling, Data Science, and Finite Element Analysis (FEA)

integrates multi-domain measurements and estimates internal mechanical or physiological states that are difficult or impossible to measure directly.

D6.1 Predictive Biomechanical Modeling

- Musculoskeletal modeling
- OpenSim-based inverse kinematics and inverse dynamics workflows
- Finite element analysis (FEA)
- Multi-body dynamics simulations
- Subject-specific modeling
- Image-based modeling
- Biplane videoradiography from Domain 1
- Physics-informed neural networks

Representative citations: (Abdullah et al., 2024; Delp et al., 2007; Hicks et al., 2015; Pandy, 2001; Pizzolato et al., 2017; Schiebl et al., 2022; Maas et al., 2012; D. Lin et al., 2023; Huang et al., 2018; L. Li et al., 2017; Weng et al., 2025; Xue et al., 2021; Nikpasand et al., 2023).


D6.2 Data Science, Signal Processing, and Machine Learning

- Signal processing methods
- EMG decomposition
- Time-frequency analysis
- Wavelet transforms
- Independent component analysis
- Filtering
- Event detection
- Synchronization of multimodal signals
- Machine learning and data-driven approaches
- Automated image segmentation
- Pose estimation
- Physiological signal classification
- Response phenotyping
- Clustering
- Prediction
- Statistical modeling

Representative citations: (Ghezelbash et al., 2024; Kijowski et al., 2020; Mathis et al., 2018; Weber et al., 2021; Bialosky et al., 2018).


Domain 7. Sensory, Psychophysical, and Contextual Assessment

characterizes sensory processing, pain modulation, autonomic reactivity, psychological state, expectations, real-world symptom dynamics, and other contextual factors that shape individual response.

D7.1 Sensory Thresholds, Pain Modulation, and Descending Inhibitory Control

- Standardized Quantitative Sensory Testing (QST) protocols
- Thermal detection and pain thresholds
- Mechanical detection thresholds
- Vibration detection thresholds
- Pressure pain thresholds
- Wind-up or temporal summation of pain
- Pressure algometry
- Conditioned pain modulation (CPM)
- Temporal summation of pain
- Offset analgesia
- Diffuse noxious inhibitory control protocols

Representative citations: (Moman & Hooten, 2021; Rolke, Baron, et al., 2006; Rolke, Magerl, et al., 2006; Weaver et al., 2022; Bhattacharyya et al., 2023; Kinser et al., 2009; Mutlu & Ozdincler, 2015; Park et al., 2011; Coronado et al., 2012; Bialosky et al., 2014; Randoll et al., 2017; Penza et al., 2017; Vigotsky & Bruhns, 2015).


D7.2 Autonomic Nervous System Reactivity

- Heart rate variability
- Electrodermal activity
- Respiration
- Blood pressure
- Skin temperature
- Pupillometry

Representative citations: (Amin & Faghih, 2022; Bianchi et al., 2022; Electrophysiology, 1996; Hart et al., 2017; Mathôt, n.d.; Shaffer & Ginsberg, 2017; Araujo FX et al., 2019; Kovanur Sampath K et al., 2024; Alanazi MS et al., 2024; Roura et al., 2021).


D7.3 Psychometric and Ecological Momentary Assessment

- Pain intensity
- Function
- Quality of life
- Treatment expectations
- Pain catastrophizing
- Fear avoidance
- Self-efficacy
- Anxiety
- Depression
- Sleep
- Perceived improvement
- Ecological momentary assessment

Representative citations: (Anzolin et al., 2020; Bialosky et al., 2008; Bishop et al., 2011; Boehme et al., 2020; Corso et al., 2019; Kutschera et al., 2021; Panchal & Hendrick, 2023; S. M. Smith et al., 2020; Uddin et al., 2021).


Domain 8. Mechanistic Perturbation and Neuromodulation Methods

provides tools for perturbing specific neural, sensory, vascular, or biomechanical components of the system to strengthen causal inference.

D8.1 Biofeedback, Virtual/Augmented Reality, and Mechanical Perturbation Paradigms

- Biofeedback systems
- Visual, auditory, and haptic feedback
- Real-time force feedback
- Quantifiable soft tissue manipulation systems
- Virtual reality and augmented reality systems
- Mechanical perturbation and vibration paradigms
- Local vibration
- Tendon vibration
- Stochastic resonance approaches
- Whole-body vibration

Representative citations: (Calderone et al., 2025; Giggins et al., n.d.; Gudavalli et al., 2015; Bhattacharjee A et al., 2021; Bhattacharjee et al., 2023; Best et al., 2014; C. Haas, Butterfield, Zhao, et al., 2013; McCrum et al., 2022; Simon et al., 2024).


D8.2 Pharmacologic Modulation and Non-Invasive Brain Stimulation

- Pharmacologic modulation
- Nerve blocks
- Local anesthetic blocks
- Non-invasive brain stimulation
- Transcranial magnetic stimulation
- Transcranial electrical stimulation
- Repetitive TMS

Representative citations: (Edinoff et al., 2021; Kage et al., 2021; Moretti et al., 2024; Christiansen et al., 2018; Dishman & Burke, 2003; Haavik et al., 2016, 2018; Molina-Álvarez et al., 2022).
